# Mobile Health and Wellness Project: A binational collaboration of frontline health services to the Latino population in the United States in times of COVID-19

**DOI:** 10.3389/fpubh.2022.1022772

**Published:** 2023-01-09

**Authors:** Cecilia B. Rosales, Hilda Dávila Chávez, Michael A. Flynn, Juanita Lara, Isaura Angélica Lira Chávez, Leonardo Olivares Marín, Alejandra Romero Rangel, Ricardo Hirata Okamoto, Maria Gudelia Rangel Gómez

**Affiliations:** ^1^Division of Public Health Practice and Translational Research, Mel and Enid Zuckerman College of Public Health, University of Arizona, Phoenix, AZ, United States; ^2^Instituto de los Mexicanos en el Exterior, Mexico City, Mexico; ^3^Universidad Nacional Autónoma de México, Mexico City, Mexico; ^4^National Institute for Occupational Safety and Health, Washington, DC, United States; ^5^Comisión de Salud Fronteriza México-Estados Unidos, Tijuana, Mexico; ^6^Keisen Consulting Inc., Mexico City, Mexico; ^7^El Colegio de la Frontera Norte, Tijuana, Mexico

**Keywords:** COVID-19, access to health care, preventive health care, Mobile Health Units, health initiative

## Abstract

Hardly reached communities in the United States greatly benefit from collective efforts and partnerships from Community Based Organizations, Health Institutions and Government Agencies, yet the effort to engage in this collaborative effort is minimal and funding to support these projects is lacking. The COVID-19 Pandemic exacerbated on a national scale what many vulnerable communities experience regularly; difficult access to basic medical care, information and support. In an effort to directly engage with community organizations and curb the infection rate of the COVID-19 virus within vulnerable communities, the US Centers for Disease Control and Prevention (CDC) launched its first targeted effort to partner directly with community based organizations. This article will highlight the first pilot year of activities and key results of COVID-19 education and vaccination efforts by the Mobile Health and Wellness project. This is a fleet of 11 Mobile Health Vehicles managed by the Mexico Section US-Mexico Border Health Commission in partnership with Alianza Americas, Latino Commission on AIDS, and the CDC, targeting Latino, Immigrant and rural communities across the US.

## 1. Introduction

COVID-19 has exposed the challenges and vulnerabilities that health systems all around the world face. The pandemic corroborated the disparities and inequities we've known vulnerable communities have always had to overcome when trying to access health care services. In the United States, Black and Latinx communities have had to deal with some of the most harmful health and economic effects left by this contagion.

According to the Economic Policy Institute, Latinx workers have suffered greater economic hardships than their white counterparts. Even prior to the pandemic, Latinx workers faced low wages and precarious health conditions, in addition to lower access to health care (1).

Initially the impact of COVID-19 in regard to death rates for Latinx and white non-Latinx populations seem similar (39–35 deaths per 100,000). However, the results widen as age groups are considered. For example, Latinx children ages 0–14 are 3.3 times more likely to die from COVID-19 than white children from the same age group. Also, Latinx youth ages 15–24 are 6.1 times as likely to die than those considered white (1).

There are also several underlying economic factors that exacerbate the solvent effects of COVID-19 on the Latinx community, as most of these workers were economically insecure and without equal access to health care long before the pandemic even started. Most of the issues that Latin Americans face can be tracked to the structural and institutional racism in the country (2).

As the COVID-19 pandemic continues, it has become apparent the Latinx community is one of the most affected communities amongst the population. According to data from New York City -an early epicenter of the coronavirus infection-, immigrants are over-represented in the front lines of health care, transportation, agriculture, food production, and sanitation (3, 4).

A large percentage of Latinx immigrant workers are not able to work from home. Even though they tend to play essential roles in the American economy, they often lack access to personal protective equipment (PPE) and fear accessing COVID tests or medical services. The Economic Policy Institute documented that Latinx immigrants are also not able to social distance at home or at work. They lack access to viable and trusted information due to language and cultural barriers, which generates fear of being stigmatized. As a consequence, the pandemic has also exposed the lack of safety measures in the workplace (1).

This widely known, but rarely addressed, context affecting communities of color and immigrants, called for partnerships that bridged the guidance of scientific and health professionals with organizations that already established trust with communities at most risk. Thus, the Centers for Disease Control and Prevention (CDC) launched the “Improving Clinical and Public Health Outcomes through National Partnerships to Prevent and Control Emerging and Re-Emerging Infectious Disease Threats” grant opportunity, to work closely with national partners.

The Mexican section of the United States-Mexico Border Health Commission (USMBHC) in partnership with Latino Commission on AIDS (LCOA), participated in this Project led and coordinated by Alianza Americas (AA). This trio collaborate and join forces to curb the current infection rate within vulnerable communities and contribute to the improvement of national efforts to address re-emerging infectious diseases.

The Mexican Section of the USMBHC operates a fleet of Mobile Health Units (MHU). These 11 mobile vehicles launched in 2016 are located in: Chicago, Dallas, Denver, Las Vegas, Los Angeles, Miami, New York, Orlando, Phoenix, Raleigh, and Tucson. The MHU are the only model of care that offer preventive health care services on priority health issues to the Latinx population in remote communities experiencing difficult access to health and public health services. Key services provided include: orientation and counseling, basic health screenings, referrals, and vaccines. Care is culturally and linguistically adapted, free, and accessible regardless of insurance coverage or immigration status. It was within this context that the Mexican Section of the USMBHC, with the MHU, began working closely with the CDC to reduce the impact and stop the spread of COVID-19, as well as other potential disease threats (5, 6).

This article describes the efforts done by the Mobile Health Units during the first stage of this Project from February to September 2021. It aims to highlight key outcomes on COVID-19 education and vaccination efforts, as part of MHU's contribution to this Project.

### 1.1. Mobile Health and Wellness Project

The specific aim of the grant is to improve health promotion and response activities to limited English proficiency (LEP) Latinx essential workers, as well as their families and their communities. A key strategy of this effort includes strengthening critical partnerships with community-based organizations (CBOs). It also aims to develop culturally and linguistically tailored programs and practices to facilitate the dissemination of information, testing, contact tracing, vaccination, and the development of healthcare strategies as a direct response to health emergencies such as the COVID-19 pandemic.

The project consists of three strategic initiatives and their corresponding activities: disseminate and adopt, inform and adapt, and target and train. Each strategy proposes a series of short-term results, where it seeks to increase knowledge on COVID-19 and facilitate access to the COVID-19 vaccine, for both the target population and essential workers. They also seek to create alliances with local city and state health departments and with non-governmental organizations. The purpose is to carry out community events such as health fairs, educational programs, and outreach.

The first strategy, *disseminate and adopt* seeks to support the CDC in the dissemination and adoption or implementation of COVID-19 guidelines to curb the infection rate. It includes vaccination readiness plans, clinical guidelines, and best practices for the prevention and control of emerging and re-emerging infectious diseases. The strategy aims to work with limited English proficiency Latinx essential workers, their families, and communities.

The activities developed for this strategy include digital dissemination of existing and adopted resources *via* social media. The information sources are original infographics, video, and audio resources in alignment with CDC guidelines, testimonials, and public radio and television spots. Other activities are the participation and organization of local community and health events, as well as the distribution of cultural and linguistically adapted printed materials. Lastly, it looks forward to establishing partnerships with local governments and agencies.

The second strategy, *inform and adapt*, seeks to inform, and support CDC in the development and adaptation of guidelines, tools, and best practices. Including collecting information and communicating effectively. The activities embrace feedback sessions on best practices and dispelling myths and addressing misinformation and disinformation. It also incorporates partnerships with state and city departments of health.

The third strategy, *target and train*, seeks to engage frontline personnel and lead training in CDC best practices for the broader workforce supporting the prevention and control of emerging and re-emerging infectious diseases. Target guidance and tools to better reach communities and populations at increased risk for infectious diseases and reduce disease spread in targeted workplaces or settings.

The activities for this strategy are the recruitment and training of community health workers, volunteers, interns, and students. Also, the promotion of important relationships with state and local health departments to support vaccination activities.

The MHU implemented these efforts as part of their core activities, many of which were already in practice. Targeted outreach, building trust, individual and personalized orientations are among the activities in action. Access to educational material and viable and trusted information that is culturally and linguistically sensitive is key to successfully providing a quality service that is informative and impactful.

### 1.2. Target population

Through the Mobile Health and Wellness project, community health initiatives are implemented as a first response to the prevention of emerging and re-emerging infectious diseases, as well as non-communicable diseases, mental health initiatives, among others. The project contributes by improving access to basic health services to vulnerable Latinx communities. Significant part of this population is in a geographically hard to reach areas and with difficult access to these and other basic services.

The main characteristics of this population, includes low income, limited English proficiency, prevalent barriers to access health services, a remote or rural locations, and rampant marginalization. These represent some of the most frequent barriers to healthcare for the Latinx immigrant population in the United States, and directly affect their overall wellness (5, 7). Additionally, the most common employment for this population includes agriculture, home and office cleaning, construction, and industrial factory work. Moreover, more than 70% of this community is un-or under insured (5).

The MHW offers a vast national network and infrastructure, with the capacity to link some of the most vulnerable communities in the United States with local providers who can provide medical care, together with national public health organizations dedicated to improving access to health services and eliminating health disparities.

## 2. Methods: Process measure indicators

For the preparation of this article, qualitative methods were used based on reports directly related to the project, such as the project Logic Model, monthly progress reports, and the cumulative final report.

From the strategies involved in the logical model: disseminate and adopt; inform and adapt; and target and train, its components were reviewed based on the indicators of population served, preventive health services provided, development of educational material and dissemination of information, and recruitment and training. Rollout of activities based on the logic model were implemented by the MHU and tracked using newly developed reporting tools and an online database, tracking the number of individuals reached and services provided.

## 3. Results

The main results of the project are described below, based on the committed indicators, in each of the strategies that make up the logical model.

### 3.1. Disseminate and adopt

The MHU offered a total of 1,535,771 services to 245,541 people during February–September 2021 (Table 1). The services include health orientations, basic health screenings, vaccines, and referrals to health services. They target priority health issues affecting the migrant community, highlighting COVID-19 specific services (Table 2) that provide relevant health care topics in the face of the COVID-19 pandemic.

**Table 1:** Basic services provided by the Mobile Health Units, February–September 2021.

**People receiving services**	**245,541**
Services provided	1,535,771^(a)^
Orientation and information	1,442,181 (93.9%)
Health screenings conducted	38,737 (2.5%)
Vaccines given	54,625 (3.6%)
Health referrals	228 (0.01%)

^(a)^People could attend >1 services; therefore, number is higher than total people.

Source: MHW project, integrated logic model and cumulative report February–September 2021, extracted from database *Sistema de Información Continua y Reportes de Salud de los Mexicanos en Estados Unidos* (SICRESAL-MX), from the Mexican Section of the United States-Mexico Border Health Commission.

**Table 2:** COVID-19 specific services provided by the Mobile Health Units, February-September 2021.

**Services provided**	**No**.
COVID-19 orientation and information	68,238 (65%)
COVID-19 screenings conducted	5,740 (5.5%)
COVID-19 vaccines	31,000 (29.5%)
COVID-19 referrals	13 (0.01%)

Source: MHW project, integrated logic model and cumulative report February–September 2021, extracted from database *Sistema de Información Continua y Reportes de Salud de los Mexicanos en Estados Unidos* (SICRESAL-MX), from the Mexican Section of the United States-Mexico Border Health Commission.

For the most part, the MHU focused on orientations (informative talks on various health topics), which make up 93.9% (1,442,181) of the total services provided. It is followed by the application of vaccines with 3.6% (54,625) and screenings with 2.5% (38,737) of the total. Likewise, the total referrals for this period were 228 (0.01%). Regarding COVID-19 specific services, a total of 104,991 were provided: COVID-19 orientations made up 65% (68,238), COVID-19 vaccines makes up 29.5% (31,000), 5.5% (5,740) of services corresponded to COVID-19 screenings, and COVID-19 referrals reported a total of 13 (0.01%).

These numbers reflect comprehensive preventive healthcare services to the Latinx immigrant communities in the United States, which are among the most vulnerable communities even prior to the beginning of the pandemic.

The dissemination of information included newly developed material based on guidelines and current updates from various trusted and official sources. Some of them are the CDC, Mexico's Ministry of Health, non-profit institutions, as well as the Pan American Health Organization (PAHO), and the World Health Organization (WHO). The documents include data on the early stages of the pandemic, symptoms, risk factors, preventive measures, COVID-19 testing, treatment for persons with compromised immune systems or existing preconditions, and mental health during the pandemic.

Information and credible resources provided by these institutions guided efforts to dispel misleading information on COVID-19: symptoms, level of contagion, treatment, as well as vaccines, vaccine side effects, and boosting vaccine confidence. In total, 86 educational materials about COVID-19 were developed during this timeline. These materials also included information on the new variant of the virus, COVID-19 vaccine updates, as well as mental health in times of the pandemic.

All materials and messaging were shared by the MHU on social media platforms and during community events, both virtual and in-person. The dissemination of information on social networks (Facebook, Twitter, Instagram, and YouTube), yielded the following results: reach-341,860; reactions-9,890; comments-3,089; and shares-1,741.

In addition to these platforms, other technology was used such as Facebook live, open virtual forums on frequently asked questions, videos to eradicate the myths of the vaccine, and publications of COVID-19 vaccination days. Also, there was the dissemination of photographic evidence of people being vaccinated, infographics of preventive measures, and video testimonials. These other forms of outreach yielded: 1,006,410 reached on Television and 427,870 reached on radio stations.

Additionally, 355,500 brochures with COVID-19 information were printed. They highlighted spread and prevention, myths and facts, vulnerable population, vaccines, and mental health during the COVID-19 pandemic. All of this material was distributed during in-person events.

The MHU held 51 community events known as *Ferias de Salud* (community health fairs or events). Some of these events were carried out exclusively by the MHU, while others were planned with the assistance of local governments, as well as other community organizations. During these events, special attention was given to orientation and counseling in efforts to provide educational resources on priority health issues, screenings, COVID testing, referrals, and COVID and Influenza vaccines. In total 248 events and/or targeted activities were carried out to promote vaccination in COVID-19 confidence and administration.

### 3.2. Inform and adapt

The MHU documented key myths and misleading information that users shared concerning COVID-19 and vaccines, as well as efforts made to dispel and correct common misconceptions. Efforts included, sharing statistics of people infected with COVID-19, raising awareness and emphasizing the positive outcomes of getting vaccinated. Also, having medical professionals and pharmacists available during vaccination events to address any doubts and concerns.

The MHU generated 24 best practices specific to COVID-19 related issues, most of which were focused on process improvement, better procedure or method, interaction with other institutions, and data analysis for agile decision making. These best practices are proven to support efforts and functionality and easily replicated by other agencies.

### 3.3. Target and train

Community involvement and engagement are vital to successful dissemination of information and services. There were 187 recruitment events held where 1,585 volunteers (80%), students (19%), and interns (1%) were recruited. Of which, 1,458 were trained on priority health topics, especially on COVID-19 and the vaccine.

Mental health issues during the COVID-19 pandemic added a layer of specialized services needed. The MHU health promoters or *Promotoras*, received specialized training and support in self-care from the Faculty of Psychology of the National Autonomous University of Mexico (UNAM for its acronym in Spanish). Currently, MHU *Promotoras*, are collaborating with the stage of implementation of care through the screening, evaluation, management, and monitoring of high-risk cases in mental health during the COVID-19 pandemic. It is from these initial screenings that *Promotoras* can identify persons experiencing high levels of stress and in need of first-level psychological care services.

Subsequently, the extended network of allies specializing in mental health services facilitated access to second or third level care services. As well, the MHU offered additional social services, in response to the mental health needs of the Latinx community during the COVID-19 pandemic. These specialized mental health services were provided to 76 participants treated during the height of the COVID-19 pandemic. Of these, 61 individuals were referred to specialists from UNAM. They received culturally and linguistically adapted psychological care through telemedicine; 45.45% continue to receive this care. Anxiety and stress were among the most diagnosed conditions, followed by substance use, violence, and depression.

*Promotoras* providing these services received the “Taking care of my mental health: Skills for the management of emotions in the context of COVID-19”. These trainings, in which mental health issues were addressed and strategies were implemented, allowed the *Promotoras* to build skills in emotional management, behavioral rehearsals, and receive feedback on their participation and performance.

It is also important to highlight the 301 local alliances made, 66 of which were exclusively for vaccination purposes. These were made with local health departments, religious and educational centers, and community organizations. They not only carried out COVID-19 vaccination events but were instrumental in the distribution of educational materials to communities that struggle to trust and believe mainstream media sources.

Continued efforts included partnerships with Consulates from different Latin American countries. This is a key component to reach the many mixes status Latinx families that include both US and foreign citizenship, residents, visa holder, DACA recipients and non-status immigrants.

Alliances with State and Local Health Departments allow for the healthcare network in the country to be more inclusive and truly reach the hard-to-reach population.

## 4. Discussion

The Mobile Health and Wellness Project in collaboration with community organizations Alianza Americas, Latino Commission on AIDS, and the CDC, is a threefold strategy to develop, practice and revisit effective outreach efforts and build trust with hardly reached communities. The Project has proven to be an impactful program when collaboration is the core ingredient that drives the collective effort. This includes collaboration at the governmental level, academic institutions, community-based organizations and the community level *via Promotoras de salud*, volunteers and students at all levels (high school, college, and graduate students).

The first phase of the project (pilot phase) concluded in October 2021. As the Mobile Units continue to participate in the second phase of the project, the partnerships will continue to grow and strengthen, as well as the bond with the Latinx communities within the cities in which these mobile units operate. The MHU will continue to provide services and health education through the creation of educational materials on COVID-19 and other priority health issues, utilizing social media posts, videos, radio and TV spots, posters, webinars, among others. This will potentially encourage and motivate individuals to take care of their own family health, their families and community.

The COVID-19 virus will be among us for many more years even after the mask and distancing restrictions have been lifted and the vaccines become part of annual rituals to keep us healthy. It is vital to keep educating and providing information in order to keep vulnerable communities safe and prepared for any possible future outbreaks. However, the Mobile Health Units care model, aims to provide preventive services (guidance, screenings, vaccination, and referral), where in addition to COVID-19, other priority health issues are addressed, including HIV/AIDS, chronic degenerative diseases, mental health, cancer, healthy lifestyle, respiratory diseases, health promotion, among others.

## Data availability statement

The data analyzed in this study is subject to the following licenses/restrictions: The database belongs to the United States-Mexico Border Health Commission, and is under confidentiality protection laws. Requests to access these datasets should be directed to US-Mexico Border Health Commission, grangel2009@gmail.com.

## Author contributions

IL, LO, and AR: first draft and data analysis. CR, HD, MF, JL, RH, and MR: revision and final draft. All authors contributed to the article and approved the submitted version.

## References

[1] Gould E Perez D Wilson V. Latinx Workers—Particularly Women—Face Devastating Job Losses in the COVID-19 Recession. (2020). Available online at: https://www.epi.org/publication/latinx-workers-covid/ (accessed October 08, 2022).

[2] Roque L Zamarripa R. Disproportionate Health and Economic Impacts From COVID-19. Washington, DC: Center for American Progress (2021).

[3] Ross J Diaz CM Starrels JL. The disproportionate burden of COVID-19 for immigrants in the Bronx, New York. JAMA Intern Med. (2020) 180:1043–4. 32383754 10.1001/jamainternmed.2020.2131

[4] Strathdee S Abramovitz D Harvey-Vera A Vera C Rangel G Artamonova I. Prevalence and correlates of SARS-CoV-2 seropositivity among people who inject drugs in the San Diego-Tijuana border region. PLoS ONE. (2021) 16:e0260286. 34807963 10.1371/journal.pone.0260286 PMC8608290

[5] Rangel Gómez MG López Jaramillo AM Svarch A Tonda J Lara J Anderson EJ. *Together for* health: an initiative to access health services for the Hispanic/Mexican population living in the United States. Front. Public Health. (2019) 7:273. 31608268 10.3389/fpubh.2019.00273 PMC6769102

[6] Yeager S Abramovitz D Harvey-Vera A Vera C Algarin A Smith L. Factors associated with COVID-19 testing among people who inject drugs: missed opportunities for reaching those most at risk. medRxiv. (2022). 35473678 10.1186/s12889-022-13273-y PMC9042668

[7] Strathdee S Abramovitz D Harvey-Vera A Vera C Rangel G Artamonova I. (2021). Correlates of COVID-19 vaccine hesitancy among people who inject drugs in the San Diego-tijuana border region. Clin Infect Dis. (2022) 75:e726–33. 35024825 10.1093/cid/ciab975 PMC8690110

